# Recognition of unfamiliar predators in domestic horses through only visual predator cues

**DOI:** 10.1371/journal.pone.0349298

**Published:** 2026-07-15

**Authors:** Rachel Hofacker, Natalie Sebunia, Jessica Pihlblad, Zeynep Benderlioglu

**Affiliations:** 1 Department of Evolution, Ecology, and Organismal Biology, The Ohio State University, Columbus Ohio, United States of America; 2 Current Affiliation: College of Veterinary Medicine, Ohio State University, Columbus Ohio, United States of America; 3 Current Affiliation: Cornell University, College of Veterinary Medicine, Ithaca New York, United States of America; 4 Current Affiliation: College of Veterinary Medicine, The University of Arizona, Tucson, Arizona, United States of America; Long Island University - CW Post Campus: Long Island University, UNITED STATES OF AMERICA

## Abstract

Acoustic, olfactory, and visual predator cues trigger various adaptive responses among a variety of prey species even when they are under human protection since birth. Previous studies have found that domesticated animals show increased vigilance, stress, flight, and aggregation behaviors in response to predation threats. These investigations, however, did not directly test visual threatening cues alone in animals that communicate more with body language than vocalizations while considering individual temperament and social status. The current study aims to address this issue. Eighteen horses housed at the Ohio State University Equine Center with mixed age and sex were the subjects of the experiments (mean age = 7 years). The horses were shown affiliative and aggressive behaviors of a pack of wolves (unfamiliar predator) and grazing wombats (unfamiliar non-predator) on a projector without any acoustic cues while their reactions were recorded with an equine heart monitor and camera. Data were also collected on assessments of anxiety/fear, social dependency, and social status in the herd. Results showed that domestic horses distinguished an unfamiliar predator from an unfamiliar non‑predator based on visual cues alone, showing significantly higher heart rate (HR) responses to wolf videos than to wombat videos (*P* = 0.0022). HR also increased relative to baseline during wolf videos (*P* = 0.0005), whereas HR during wombat videos did not differ from baseline (*P* = 0.28). There were no significant differences in HR responses to affiliative vs. aggressive behaviors displayed by wolves (*P* = 0.4033). The age of the horses was negatively associated with fearfulness (*P* = 0.0048) and social dependency (*P* = 0.0076). Male horses showed a more heightened HR to unfamiliar predator cues compared to females (*P* = 0.0088). High social status was associated with an increased HR response to unfamiliar predator stimuli (*P* = 0.033) but was unrelated to control stimulus HR (*P* = 0.26), baseline HR (*P* = 0.32), fearfulness (*P* = 0.26), social dependency (*P* = 0.85), age (*P* = 0.42), and sex (*P* = 0.61). Lateralized gaze revealed a near total absence of left eye viewing across all stimuli, with no difference between right gaze and binocular look durations for wolves (*P* = 0.83). In contrast, horses showed a significant binocular preference over right-gaze when viewing wombats (*P* < 0.0001). These results demonstrate that, based on visual cues alone, horses exhibit physiological alertness toward potential predatory threats while responding to non-threatening stimuli with exploratory gaze behaviors.

## Introduction

Domestic horses, *Equus caballus*, are prey animals that have long been under the protective care of humans since their domestication about 5,500 years ago [1,2]. Although their main natural predator, the grey wolf, *Canis lupus* has been reintroduced in North America and Europe [3], domestic horses are currently under little to no predation threat and are unlikely to encounter wolves while in human care.

Adaptive behaviors in response to predators in horses and most prey species appear to remain intact, however. For example, domesticated cattle and sheep show increased vigilance, flight, and aggregation behaviors in response to predation threats [4,5]. Similarly, domestic horses distinguish between threatening and non-threatening auditory cues and natural vs. unfamiliar predator vocalizations by showing increased alertness and defensive herd formations when threatened [1]. In addition, they respond strongly to loud sounds, new objects, dogs, and veterinary procedures, presumably because of evolutionarily conserved antipredatory behaviors [6].

Expanding on previous research, the primary goal of the current study was to examine whether domestic horses can visually differentiate an unfamiliar predator-a wolf, from an unfamiliar non‑predator-a wombat, using video stimuli. We specifically asked if domestic horses would still react to the sight of a pack of wolves engaging in aggressive or affiliative behaviors in the absence of auditory or olfactory cues. We hypothesized that horses would be able to differentiate a group of wombats from a pack of wolves and show increased hypervigilance and alertness to the sight of the latter marked by an increased heart rate and overt behavioral reactions, such as movement of the ears, tail swishing, and nostril dilation, behaviors associated with heightened stress or hypervigilance [7,8].

A secondary goal of the study was to assess whether horse temperament, including fearfulness and social dependence, and the social status in the herd would affect horse cognition and physiological responses to potentially threatening stimuli. Temperament is defined by the tendency for an animal to express the same behavior in various situations and may be manifested as willingness to explore, being social or anxious, or displaying aggression and dominance [9]. We hypothesized that fearfulness and general nervousness when handled, social dependency and anxiousness when separated from other horses, and lower social status in the herd would be positively associated with an increased heart rate upon exposure to wolf videos. Given prior evidence that fearful and lower-ranking horses experience elevated stress due to frequent conflict and displacement during feeding and other herd interactions [10], we expected this outcome.

## Materials and methods

Twenty animals of mixed age and sex at the Ohio State University Equine Center were initially used in the experiments. Two horses were later dropped from the study, because they did not look at the projector screen during any part of the stimulus presentation. As a result, 18 horses (mean age = 7 years, SD = 6; N _females_ = 12; N _males_ = 6) constituted the final sample. All males were geldings. The housing, food provisioning, and animal welfare and care were provided by the OSU Equine Center staff. All experiments were video recorded. Each horse was tested only once.

All experiments were conducted during morning hours until 2:30 pm to ensure that scheduled feedings in the afternoons did not confound the trials. Testing took place in Summer/Early Fall of 2021.

The study was approved by the OSU’s animal care and use committee IACUC with Protocol No: 2021A00000072.

### Stimuli

**Fig 1:**
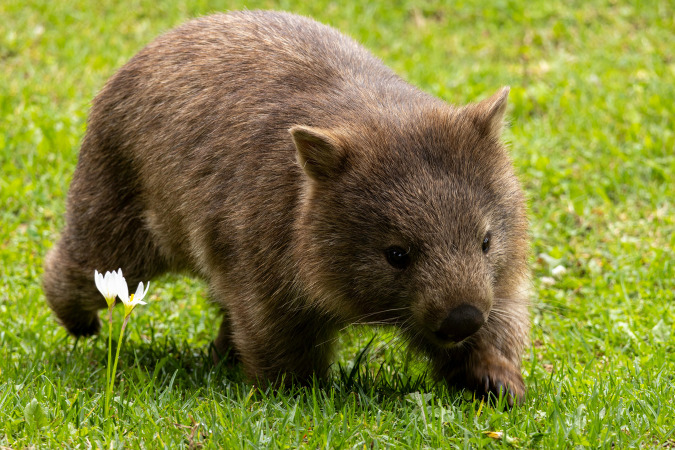
Representative image of the non-predator control stimulus. This image of a common wombat (*Vombatus ursinus*) is for illustrative purposes only and is similar, but not identical to the non-threatening control stimulus used in experimental trials. Image credit: Steve Burcham via Pexels permitted for free use, distribution, and modification.

**Fig 2:**
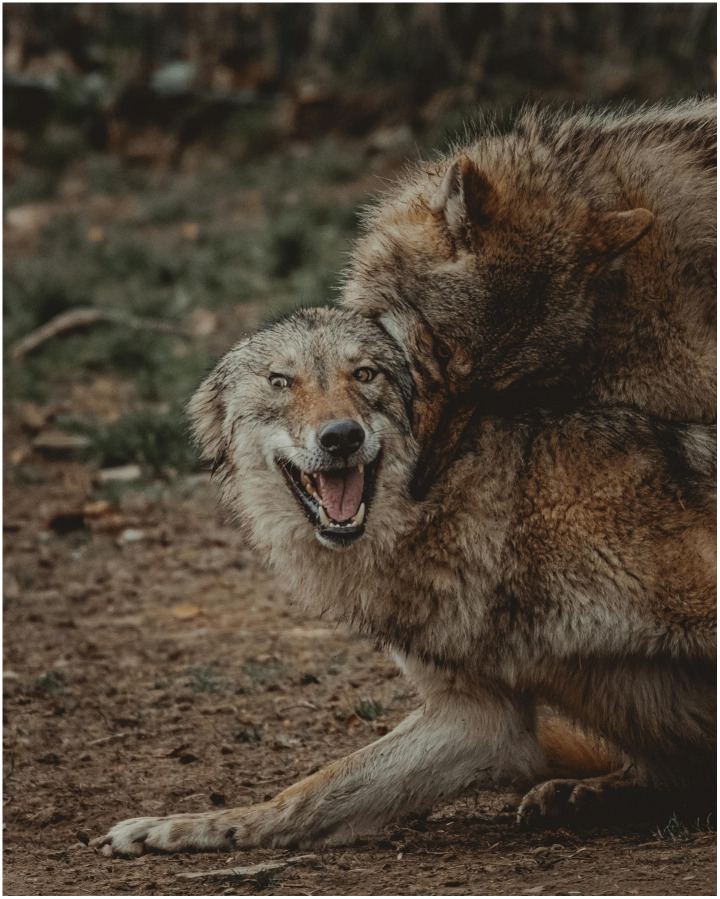
Representative image of the predator stimulus depicting aggressive behavior. This image is for illustrative purposes only and is similar, but not identical to the video segments of fighting wolves (*Canis lupus*) used to elicit anti-predator responses during the study. Image credit: David Selbert via Pexels permitted for free use, distribution, and modification.

**Fig 3:**
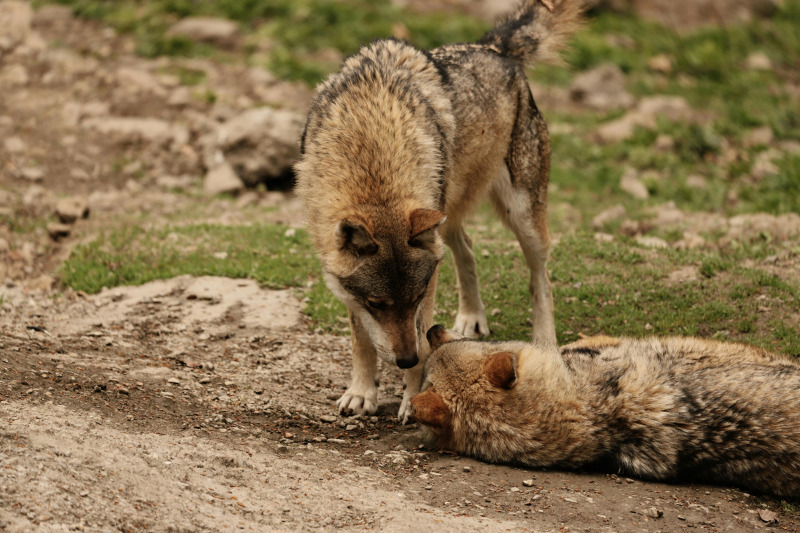
Representative image of the predator stimulus depicting social grooming. This image is for illustrative purposes only and is similar, but not identical to the video segments of wolves (*Canis lupus*) grooming one another that were used to elicit anti‑predator responses during experimental trials. Image credit: Christina & Peter via Pexels permitted for free use, distribution, and modification.

Each horse was shown a 60‑second video composed of three consecutive 20‑second segments, presented without gaps or blank screens. All footage was obtained from publicly available sources and edited to produce standardized stimulus segments. The first segment served as the control stimulus (20 seconds; Fig 1) and depicted three free‑ranging wombats grazing. The second segment showed aggressive interactions among six wolves, including biting, limb‑pulling, chasing, and fighting (20 seconds; Fig 2). The third segment displayed affiliative behaviors among five wolves, such as licking, nibbling fur, and mutual grooming (20 seconds; Fig 3). A 60‑second total duration was selected based on prior work indicating that this length is sufficient to elicit a stress response in domestic horses, and the animals lose interest in stimuli beyond the one-minute period [7].

The sequence for the 2nd and 3rd videos was counterbalanced to control for the order of presentation, because seeing aggressive videos first could potentially affect the subsequent behavior of the horses. The first stimulus was always the control (wombats grazing) against which we compared horses’ reactions to threatening stimuli from a natural predator and assessed whether horses could differentiate affiliative and conflict behaviors in unfamiliar predators. The control condition also ensured that the horses wouldn’t just react to moving scenes and unfamiliar animals. Half of the horses were randomly assigned to the sequence wombat → aggressive wolves → grooming wolves, and the other half to wombat → grooming wolves → aggressive wolves. Each horse was tested on only one group.

#### Heart rate measurements.

Each horse was fitted with a Polar Equine Heart Rate Monitor for Trotters (Polar, USA) to record heart rate during the baseline and stimulus presentations. The monitor was wirelessly connected to the Polar Equine App (downloaded from Google Play) on the first author’s Android phone, which provided continuous beat‑to‑beat (R–R interval) tracking. The R–R interval data were processed using the Heart Rate Variability Logger application (A.S.M.A., Heart Rate Variability Logger (Version 5.0.7) and exported to Excel for calculation of mean heart rate during the acclimation period and each stimulus.

After the monitor was fitted, the experimenter remained near the horse without interaction for 4 minutes to minimize any handling‑related arousal and rule out any anxious behavior. The horse was then led into the testing stall and allowed to acclimate for an additional 4 minutes; heart rate recorded (HR) during this period served as the baseline. HR was then continuously collected throughout the three video presentations. Comparing baseline HR to stimulus HR allowed us to confirm that the horses’ physiological responses during the trial were within a normal range and that any increases or decreases in HR were stimulus related rather than due to residual arousal from handling or stall entry. To ensure precise alignment between HR data and stimulus onset, timestamps were matched to the corresponding segments of the three video stimuli, aided by video recordings of each trial.

#### Projector and experimental setup.

**Fig 4:**
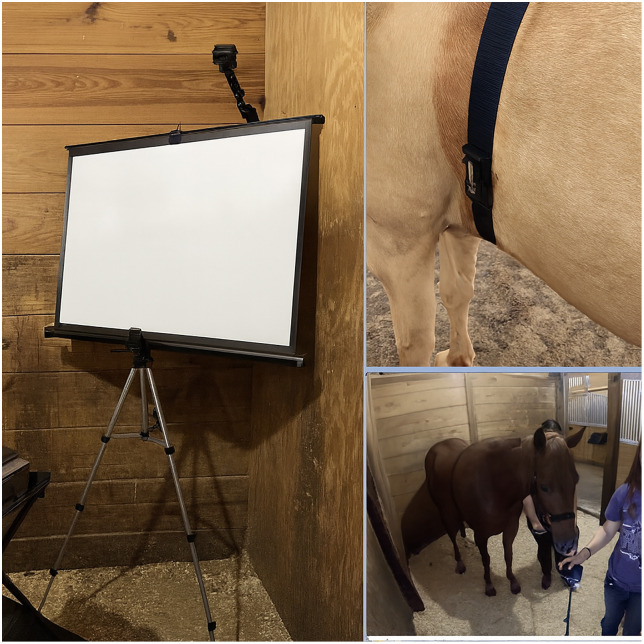
Experimental Setup a) A domestic horse with the HR monitor (top right); b) another subject (bottom right) with a handler and one of the researchers setting up the projector and videos. The handler will turn her back to the stimulus during videos while still holding the horse through the animal’s harness and the researcher will leave the stall to operate the equipment remotely before the video presentations; **c)** Projector screen setup (left) in an experimental stall with a GoPro on top for recording. All photographs were taken by the author (Z. Benderlioglu) and are licensed under CC BY 4.0.

Each horse was restrained with a lead rope held by a handler standing on the horse’s left side, with her back turned to the screen in the test stall. The handler was blind to the experimental conditions. The projector screen was placed in a corner against the stall wall, and horses were led forward until they stood directly in front of the 34.2″ W (86.87 cm) × 26.7″ H (67.82 cm) display (Fig 4). A GoPro HERO6 Black action camera (GoPro Inc., San Mateo, CA) mounted on top of the screen recorded each horse’s behavior throughout the trial.

Because the horses were periodically housed indoors or brought into stalls, including those used in the experiment for routine veterinary procedures, farrier work, and educational activities, all subjects were familiar with both the stalls and the general layout of the barn, making the test environment familiar in structure and context. During testing, horses did not have visual contact with conspecifics, although they remained close enough to hear one another.

#### Behavioral measures.

We conducted a staff survey to assess the temperament and social status of the horses in the herd. Five Equine Center staff members completed the evaluations. All five individual members were present during the full year of the study, including protocol design and the 6‑month experimental phase, and were therefore well acquainted with each horse’s typical behavior and management routines**.** Previous studies have found a strong correlation between caretakers’ subjective rankings and the behavioral data suggesting that experienced individuals can reliably assess dominance hierarchies and social status through daily interactions and observations [11–13].

The Equine Center staff provided short evaluations of the horses’ temperament and social status with score sheets. We then summed these scores to generate a total index for each horse on fearfulness, social dependency, and status separately. Specifically, horses were assessed for fearfulness and general nervousness when handled; social dependence (difficult to separate from other horses) in a familiar and unfamiliar environment; and social status within the herd. We defined social status as the tendency of individuals to consistently displace or be deferred to by herd mates, thereby gaining priority access to food, water, shelter, and preferred resting and standing areas, and influencing group movement as well as the initiation or termination of social interactions.

Moreover, several stress, hypervigilance, and alertness reactions in line with previously established behaviors for threatening stimulus were noted [7,8]. Negative stimuli result in stress-related behaviors, increased and longer recovery HR, and a left-gaze bias in horses [7,8]. Furthermore, nostril dilations are a well-established expression of pain-free stress in horses [8,14,15, but see 16]. Nostril dilations, also referred to as nostril flares, have been hypothesized to serve a physiological function by increasing air uptake during a flight response in horses [14,16], and may therefore indicate stress, alertness, or hypervigilance.

We thus noted the duration of left-gaze, right-gaze, and binocular looks, ear movement (backward or forward, only one ear moving), as well as occurrences of tail swishing, head bobbing, nostril dilation, defecation, and urination during the presentation of stimuli. We used a behavior analysis software, ZooMonitor [17] to record the behaviors. Two scorers (NS & JP) watched the videos to mark the observed behaviors independently. They were blind to experimental conditions and were only informed of the start and end points for scoring. An ethogram with operational definitions was developed to ensure consistent identification of each behavior. Both observers independently scored all videos. Inter‑observer reliability was calculated using percentage agreement, defined as the proportion of instances in which both observers coded the occurrence of a behavior identically. Agreement between observers was 98.1% (SD = 3.1). Given this high level of concordance in behavior occurrence, the durations of behaviors were averaged across observers for later analysis.

### Statistical analysis

All statistical analyses were performed with SAS 9.4 (SAS Institute, Inc., Cary, NC). A mixed within-subjects model (PROC MIXED) was used to compare mean HR from the 4-minute baseline to each stimulus type (wombat control, wolf-fighting, wolf-grooming), and to compare HR in the wombat control condition with that recorded during the two wolf segments. Statistical significance was assessed using multiple comparison tests. The same PROC MIXED framework was also used to evaluate the duration of behavioral measures (right-gaze, binocular looks, left-gaze, and ear movements) across stimulus types. Sex, stimulus type, and stimulus order were specified as main effects. Age, temperament scores, and social status scores were included as covariates where applicable to adjust for their potential influence on the dependent variables. To directly account for within-horse differences across trials, random intercept effects were specified at the horse level to model repeated measures. This approach ensured that individual variations in resting HR were statistically controlled through the estimation of subject-level intercepts, making the reported differences equivalent to within-individual shifts from baseline.

Least-squares means were utilized for all post-hoc comparisons. We used Kenward-Roger degrees of freedom approximation [18] for mean comparisons, because our sample had unbalanced cells; we had more females than males. PROC Mixed is particularly appropriate for unbalanced groups as it accounts for unequal samples sizes within each factor [19–21].

Behavioral occurrences were further analyzed with logistic regression analysis (PROC LOGISTIC) when outcome variables were binomial (nostril dilation, tail swishing, head bobbing, defecation and urination; coded as absent = 0, present = 1). A correlational analysis was used for the linear relationship between temperament measures with age partialled out where appropriate.

Results were considered significant when *P* ≤ 0.05 (one-tailed tests).

## Results

### Heart rate (All HR values are reported in beats per minute -bpm)

The results show that the main effect of the stimulus on HR was significant (*F*_4,68_ = 4.67, *P* = 0.0022). *Post-hoc* comparisons showed that horses showed significantly higher HR during wolf video presentations than during the wombat control videos and baseline measures (*LS Mean*: wolves–fighting = 63.9; wolves–grooming = 66.3; wombats = 55.9; baseline = 51.0, all values in bpm). Baseline HR did not differ from wombat HR (*P* = 0.28). There were no statistically significant differences in mean HR during video presentations of wolves that displayed aggressive behaviors compared to those that showed affiliative behaviors (*P* = 0.4033). Therefore, in subsequent temperament and behavioral analyses, HR measurements during wolf presentations were pooled. These values were then compared to the average HR obtained during control conditions depicting wombats. There was also no significant effect of stimulus order on HR (*F*_1,15_ = 1.91, *P* = 0.19).

**Fig 5:**
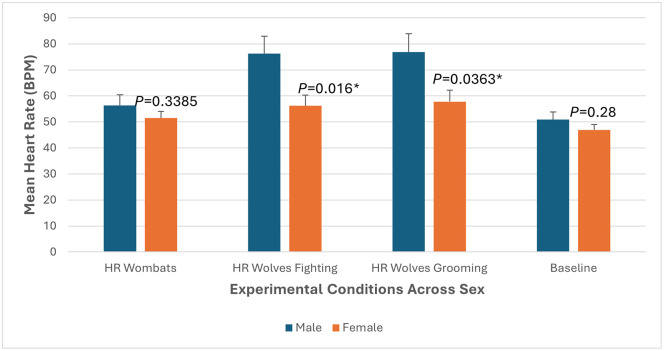
The differences in mean heart rate (HR; Beats per Minute, BPM) across experimental conditions and sex (domestic horses exposed to videos of an unfamiliar predator-wolf, or a control species-wombat). Least square means were estimated using the PROC MIXED procedure (*N*_*female*s_ = 12; *N*_*males*_ = 6). Mean HR for males were significantly higher than that of females during fighting and grooming wolf videos. There were no statistically significant differences in mean HR between males and females watching grazing wombats. Baseline HR did not differ between male and female horses. For both sexes, baseline HR was not significantly different from HR during wombat videos (LS mean comparison for males: *P* = 0.14; for females: *P* = 0.06). In contrast, baseline HR for both males and females was significantly lower than HR recorded during wolf videos (LS mean difference for males *P* = 0.0032 for wolves-fighting; *P* = 0.0025 for wolves-grooming; for females *P* = 0.0005 for wolves-fighting; *P* = 0.0008 for wolves-grooming). *significant at P<=0.05.

Both the main effect of sex (*F*₁,₁₅ = 9.07, *P* = 0.0088) and the interaction between sex and stimulus type (*F*_3,45_ = 3.45, *P* = 0.0072) were statistically significant: males showed higher HR than females during the wolf stimuli (*LS Mean*: males = 76.8; females = 54.8). No sex differences were detected during the wombat control (*LS Mean*: males = 56.3; females = 51.5; *P* = 0.34) or baseline measures (*LS Mean*: males = 50.9; females = 46.9; P = 0.28; Fig 5).

#### Temperament.

Correlational analyses indicated that neither stimulus HR nor baseline HR was significantly associated with fearfulness or social dependency (Table 1). In contrast, age was significantly and negatively associated with both fearfulness (*P* = 0.0048) and social dependency (*P* = 0.0076). We thus statistically controlled for age in the subsequent correlational analysis between fearfulness and social dependency, which revealed a significant positive relationship between the two variables (*P* = 0.0146, Table 1).

**Table 1:** Correlational relationships: temperament, age, and heart rate in domestic horses.

Variable Pair	*r*	*p*
Stimulus HR × Fearfulness	0.33	0.18
Stimulus HR × Social Dependency	0.39	0.12
Baseline HR × Fearfulness	−0.06	0.83
Baseline HR × Social Dependency	0.21	0.41
Age × Fearfulness	−0.63	0.0048*
Age × Social Dependency	−0.61	0.0076*
Fearfulness × Social Dependency (partial, controlling for age)	0.58	0.0146*

* significant at P<0.05, N=18; HR= Heart Rate.

Sex was not associated with temperament, as males and females did not differ in fearfulness (F₁,₁₇ = 0.34, *p* = 0.57) or social dependency (F₁,₁₇ = 0.77, *p* = 0.39).

#### Social status.

**Fig 6:**
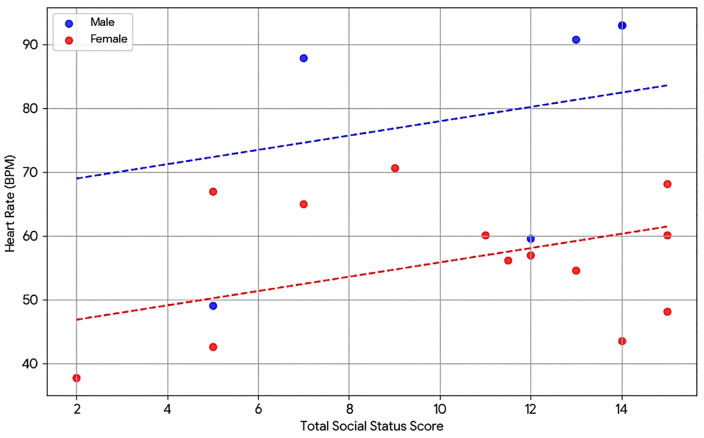
Social status score and mean heart rate (HR; Beats per Minute, BPM) during wolf stimulus presentation in domestic horses. Least square means were estimated using the PROC MIXED procedure (*N*_*females*_ = 13*; N*_*males*_ = 5). Animals with higher social status had higher HR during wolf video segments (pooled sample for both grooming and fighting wolves). The same relationship was observed for both sexes. Males had a higher HR during the wolf stimulus presentation (see also Fig 5).

Social status was not associated with either fearfulness or social dependency, as indicated by nonsignificant correlations between status and the two temperament scores (fearfulness: *r* = 0.28, *p* = 0.26; dependency: *r* = 0.05, *p* = 0.85). Social status was also unrelated to sex or age, with no significant effects detected (sex: F₁,₁₇ = 0.26, *p* = 0.61; age: *t*_*1*_ = −0.76 *p* = 0.42). In contrast, social status was positively associated with mean HR during stimulus presentations. Horses with higher social status exhibited higher HR while viewing the wolf videos (*B* = 1.62, *SE* = 0.68, *p* = 0.03326; Fig 6). However, no significant relationship was observed during the control or baseline conditions (control HR: *r* = 0.25, *p* = 0.33; baseline HR: *r* = 0.24, *p* = 0.32).

#### Overt hypervigilance behaviors.

None of the stress or hypervigilance behaviors, including left-gaze, right-gaze, and binocular looks, tail swishing, head bobbing, ear movement (backward or forward, only one ear moving with its direction noted), nostril dilation, defecation/urination was significantly associated with video stimuli HR (Table 2). Eye and ear movement results were based on duration (correlational analysis), whereas those involving defecation/urination, head, tail, and nostril movements were based on the occurrence of the behaviors (coded as yes = 1 or no = 0, logistic regression).

**Table 2:** Relationships between stimulus and eye, ear, tail, head movements, nostril dilation, heart rate, defecation/urination in domestic horses.

Variables	*r* ^*a*^	*Chi-Square* ^*b*^	*df*	*p*
Left-gaze × Stimulus HR	−0.17			0.51
Right-gaze × Stimulus HR	0.03			0.90
Binocular look × Stimulus HR	0.00			0.99
Ear forward × Stimulus HR	0.14			0.59
Ear backward × Stimulus HR	−0.06			0.83
Tail swishing		0.65	1	0.42
Head bobbing		0.10	1	0.75
Nostril dilation		0.03	1	0.85
Defecation/urination		0.02	1	0.89

*No significant association were found (p > 0.05 for all tests). N = 18, HR = Heart Rate*

a *correlational analyses on eye and ear movement results were based on duration*

b *Defecation/urination, head, tail, and nostril movement results were based on the occurrence of the behaviors (coded as yes = 1 or no = 0, logistic regression).*

We also compared the duration of right‑gaze, left‑gaze, and binocular looks toward the stimuli. Left‑gaze behavior was extremely rare: it was observed in only 3 horses during the grooming‑wolf videos and in only 2 horses during the fighting‑wolf videos, with maximum durations of 2 s and 3 s, respectively. Similarly, only 4 horses showed left‑gaze behavior toward the wombat stimulus, one for a maximum of 3 s and the others for just 1 s. Because of this extreme rarity and the resulting lack of variance in this variable, left‑gaze duration was treated descriptively and excluded from formal inferential statistical analyses for group comparisons.

In contrast, right‑gaze and binocular looks occurred frequently enough to allow robust comparison. Horses showed no right‑gaze bias when viewing wolves (*LS Means*: right‑gaze = 6.79 s; binocular = 6.65 s; *P* = 0.83). When viewing wombats, however, horses displayed a binocular preference, with significantly longer binocular look durations compared to right‑gaze behavior (*LS Means*: right‑gaze = 6.98 s; binocular = 10.31 s; *P* < 0.0001).

## Discussion

The current study assessed domestic horses’ ability to recognize aggressive and non-threatening behaviors from unfamiliar animals through presentations of video stimuli in the absence of acoustic and olfactory cues. It further investigated if the temperament and social status of the horses mitigated this assessment.

1. Heart rate responses to stimuli

Our results showed that domestic horses exhibited elevated HR when viewing an unfamiliar predator (wolf) compared with both baseline and an unfamiliar non‑predator (wombat). In contrast, HR during wombat videos did not differ from baseline. Horses also did not differ in their HR responses to the two wolf stimuli, one depicting affiliative behavior and the other aggressive behavior. Together, these findings indicate that, even without olfactory or auditory cues, horses can visually discriminate an unfamiliar predator from a non‑predator, and that this discrimination does not depend on the specific social behavior or movement displayed by the predator.

It is possible that the different colors presented in the videos constituted a limitation for horses in distinguishing wolf behavior and movement. Wombat coat color ranged from light brown to dark gray and beige, and the background color varied across videos. Furthermore, the coat colors of the wolves varied from white to almost black depending on species and individual variation. In the aggressive interactions segment of the video, five wolves had white coats and one had a dark gray coat. In the grooming videos, five wolves had coat colors ranging from white and light gray to dark gray.

Horses have difficulty differentiating certain colors from each other because of a deficiency in cone photoreceptors in their retina [22]. However, horses’ dichromatic color-perception system has a relatively strong sensitivity to luminance contrast and differences in brightness despite its restricted chromatic range [22,23]. Consequently, while they may not reliably discriminate subtle hue differences, they can distinguish variations in grayscale and contrast [23]. Moreover, horse vision may have evolved more for detection of predators from any direction than for fine-grained visual identification [24]. Given that both predator and non-predator stimuli elicited appropriate and differentiated HR responses despite variation in coat color, it is unlikely that color differences alone account for the observed patterns. Nevertheless, further research into how horses distinguish and respond to objects of various colors and intensities is needed, especially in predator and threat recognition.

One may raise the possibility that our study animals have encountered canines while kept in their pens during summer months. Also, we cannot firmly establish the birth history of the horses, although some of the experimental animals were born at the facility. These factors limit our ability to determine each horse’s prior exposure to canids. However, dogs are not allowed in the research and teaching facility where we conducted the study. OSU’s Equine Center is not open to public and requires special permission to enter. Across all of our visits between 2019 and 2025, including the study period in 2021 we did not observe domestic dogs or wild canids in or around the facility, although the presence of foxes or coyotes in the broader area cannot be ruled out. Moreover, the horses were protected by an electric fence with protected shelter when they were in large open-air pens in the summer fending off wildlife.

Our study complements previous investigations showing that horses display vigilance and fear responses to unfamiliar predator vocalizations and odors [1,10], although the responses observed in our study were limited to physiological and attentional alertness rather than overt fear. Similarly, previous studies indicate that as the distance from a predator is reduced, prey animals increasingly mobilize for action with increased vigilance, blood movement to the gross muscles, sympathetic activation of glands and smooth muscles, and higher cardiac activity marked with increased HR [25,26]. Recognizing wolves as a potential threat would confer horses an advantage in environments without human protection, and as wolf populations begin to recover in certain areas, such vigilance-based responses are likely to be retained in feral horses, and subsequently captive animals. This pattern aligns with findings in Konik polski horses, where semi-feral, forest-born individuals showed higher physiological arousal—reflected in elevated HR during human interaction and handling—compared to stable-born counterparts, even though both groups exhibited a similar fear response to a startling novel object [27,28]. Future work could examine how combinations of visual, auditory, and olfactory cues interact to elicit predator-specific responses in horses and other prey species.

2. Sex differences

Male horses had significantly higher average HR during wolf video presentations compared to females. Although this pattern could suggest higher resting HR in males, baseline values did not differ significantly between sexes, consistent with previous studies [29]. The elevated HR observed in males during predator stimuli therefore likely reflects a sex‑linked difference in reactivity rather than baseline physiological variation.

One plausible explanation is that male horses exhibit greater hypervigilance, a trait documented in free‑ranging populations [30]. This heightened vigilance may be linked to the greater social mobility of males, who, upon reaching puberty, typically leave their natal herd and either join bachelor groups or compete for access to females [30,31]. Male horses will likely be solitary for some portion of their lives, so it would be advantageous to be hypervigilant of any threats in their environment. In addition, if a male becomes the harem stallion, he should be vigilant to protect its herd as females may be taking care of the offspring. Indeed, studies show that male horses will not lead a herd during movements, but rather stay in the rear, where they can remain vigilant and scan the surroundings for any threats [32]. It should also be noted that the unequal number of males and females in our sample reduced statistical power for detecting sex effects, although robust statistical procedures were used to mitigate this limitation. Future research with larger and more balanced samples, and across different developmental stages, will be necessary to refine inferences about sex‑specific patterns of hypervigilance and physiological reactivity and HR differences.

3. Behavioral responses to stimuli

We found no significant differences in behavioral stress or hypervigilance responses regarding tail swishing, head bobbing, movement of the ears, nostril dilation, defecation, and urination between control and experimental stimuli. Specifically, horses did not show heightened stress or vigilance through such behaviors in response to videos depicting wolves fighting or grooming compared to the wombats grazing. We have thus failed to replicate previous research on increased frequency of these stress behaviors [see 7, 8, 14]. The lack of significant findings may be attributed to the shortness of the videos, with each being 20-seconds long. However, this length was sufficient to elicit a physiological arousal state as evidenced by increased HR during threatening stimuli. Perhaps horses rapidly assessed the threat, showed an initial HR increase, and then displayed muted behavioral responses when the threat did not materialize. A more likely explanation is that our visual stimuli alone were not sufficiently arousing to elicit overt behavioral reactions, even though HR measures were sensitive enough to detect differences in alertness and vigilance. This interpretation is consistent with evidence that physiological and behavioral stress responses are not always correlated in horses [33]. Field observations similarly show that mature horses do not exhibit overt behavioral signs of distress in the presence of real wolves, with free-ranging adults displaying no visible indicators of reduced welfare or panic during wolf encounters [34].

Together, these results suggest that overt behavioral responses may be context‑dependent and that the absence of visible stress behaviors does not preclude internal arousal or vigilance, which may be better captured through physiological measures. More research investigating predator responses in social animals would benefit from using different types of stimuli in group and individual settings using both physiological and behavioral measures.

Curiously, while previous literature typically associates the left eye (right hemisphere) with the processing of threatening stimuli [35,36], left‑gaze behavior was extremely rare in our study, even during wolf videos. Furthermore, the lack of significant difference between right-gaze and binocular looks during wolf video presentations suggests that no single eye dominated the inspection of predator stimuli. This pattern indicates a departure from rapid, right-hemisphere-driven threat appraisal [37]. Instead, horses appeared to rely on a combination of binocular viewing, which provides higher visual acuity and depth perception [38,39] and right eye, left hemisphere processing. The left hemisphere function is associated with sustained attention, detail-oriented cognitive processing, and the examination of novel objects [40,41], suggesting that horses may have been carefully evaluating the stimuli rather than reacting to them as immediate threats.

In contrast, a significant binocular preference was observed during the wombat control stimulus. As the wombat was consistently presented first in the sequence of video stimuli, this preference likely reflects an initial ‘exploratory’ phase. Because binocular vision facilitates fine-grained inspection [39], its use here suggests that the horses prioritized high-resolution visual analysis when first encountering an unfamiliar, non-threatening stimulus. Taken together, these results suggest that in a controlled environment, cognitive processing of novelty may override the lateralized ‘flight’ responses typically expected in predator-prey interactions.

4. Fearfulness and social dependency

Anxiety-like and fearful behaviors were positively associated with social dependency scores with age used as a statistical control. Not surprisingly, the more horses were assessed as fearful or nervous when handled, the more difficult it was to separate them from other horses in familiar and unfamiliar environments. Horses are a gregarious species that have evolved to live in social groups [34,42]. Social dependency is a beneficial trait for animals under high risk of predation. In captivity, horses do not face these same selective pressures but still retain the need for group living. Social isolation is stressful for domestic horses [42] and group housing is considered vital in horse management [43].

As domestic horses still retain variable degrees of social dependency despite conflicting human and horse interests, they also retain variable degrees of neophobia. Under human management, much time is devoted to habituating horses to new objects or procedures to reduce their response to the stimulus. Horses that are fearful of novelties are also difficult to separate from conspecifics [42]. Therefore, temperament traits such as fearfulness and social dependency are important considerations in equine handling and training. For example, introducing novel stimuli should be done cautiously, particularly in situations where a horse may already feel insecure, and socially dependent horses may require gradual, structured separation from group members—if separation is necessary at all.

5. Age and temperament

Age was negatively associated with both fearfulness and social dependency. This pattern aligns with previous research showing that older horses tend to be bolder than younger individuals, likely because they have been exposed to a wider range of stimuli over their lifetimes [44] and because social dependency typically decreases with age [45]. Therefore, considerations of the age of a horse are important, especially during exposure to novel environments or objects, or humans. Care should be taken to gradually expose young horses to novelties and ensure adequate acclimation to minimize potential stress.

6. Social status in the herd

Contrary to our predictions, individuals with higher social status had heightened HR during the presentation of silent videos showing wolves either grooming or fighting. This effect was not observed during the control condition where an unthreatening stimulus depicting grazing wombats was presented. Also, social status did not influence resting HR.

Social standing in horses is determined by agonistic interspecies interactions, control over resources, and movement of the herd [46,47]. Dominant individuals may play an important role in collective decision-making (reviewed in 48]. The hypervigilance displayed by horses with higher social status as demonstrated by heightened HR may be vital in detecting predator threats and quickly leading the herd away from the threat. Indeed, previous studies show that following individual members of the herd was positively associated with high social status and increased trust placed in the dominant individuals [48,49].

In contrast, social status was not correlated with dependency, fearfulness, age, or sex. Our findings on the lack of association between social standing and fearfulness or dependency corroborate with other studies [10,50]. Similarly, the absence of a sex effect aligns with studies showing that stallions may occupy moderate or low ranks and that females can range from dominant to subordinate [51]. The non‑linear and context‑dependent nature of social rank composition [52], and human‑determined rank allocations in captive settings [53], likely contribute to these patterns.

Previous studies have found that age is positively associated with rank with horses between the ages of 7 and 20 most likely to be dominant [47]. As horses spend more time within a given social group, their social status is expected to increase. Because our sample did not include equal representation across age groups—mean age was 7—older horses may have been underrepresented, potentially obscuring age‑related effects. Although age was statistically controlled as a covariate, a broader age distribution might evaluate age–status relationships differently. Further research with larger and more age‑diverse samples is needed to clarify how age contributes to social status in captive herds.

Another source of discrepancy in our findings regarding age and social status may result from the methods we used in determining social rank. We acknowledge that there are multiple methods for establishing social status and rank in horses [see 54]. In this study, we used a survey-based approach, relying on staff members who were most familiar with our subjects to provide their independent assessments of social status. While we recognize this is a subjective method, it is supported in literature as a viable tool when used by experienced observers [11–13]. For example, one study evaluated whether equine practitioners could accurately assess social dominance ranks among horses under their care [11]. Results showed a strong correlation between the practitioners’ subjective rankings and the behavioral data from controlled feed confrontation tests, including feeding times and agonistic or submissive behaviors suggesting that experienced caretakers can reliably assess dominance hierarchies through daily interactions and observations. Although incorporating staff surveys represents an evidence-supported method for assessing social status in horses, the use of different methodologies in assessing the rank in the herd may partially explain the null findings behind the age and social status in our study. Also, social status was assessed by caretakers without distinguishing familiar versus unfamiliar contexts, which may introduce subjectivity and contextual bias.

Several other limitations may also affect the interpretation of our findings. Birth and early-life histories were unavailable for some horses, which prevented us from controlling for developmental factors that might have influenced stress reactivity, including hemispheric lateralization, or from assessing whether individuals had early exposure to canids. Variation in wolf coat color may have affected visual salience and was not experimentally controlled. The small number of male subjects reduced statistical power for sex‑specific comparisons. These factors warrant caution and highlight areas for methodological refinement in future work. Nevertheless, the consistency of the physiological responses across stimulus types indicates that the central results—particularly the horses’ ability to discriminate predator from non‑predator cues visually—are robust despite these constraints.

### Concluding remarks

The current study’s assessments on heightened vigilance marked with increased heart rate in response to potentially threatening visual stimuli have relevance to the welfare of both horses and humans during horse-human interactions. For example, a horse might encounter a canid, such as a domestic dog, during a ride or while being transported. A defensive reaction in this context may result in injury to the horse, the rider, or bystanders. Moreover, if practitioners, caretakers, and human riders are more aware of the sex, age, temperament, and hypervigilance responses of the horses, especially when the animals are around canids, then the welfare of the horses and humans may be assured more.
